# The effect of *FcγRIIA* and *FcγRIIB* on coronary artery lesion formation and intravenous immunoglobulin treatment responses in children with Kawasaki disease

**DOI:** 10.18632/oncotarget.13489

**Published:** 2016-11-21

**Authors:** Ling-Sai Chang, Mao-Hung Lo, Sung-Chou Li, Ming-Yu Yang, Kai-Sheng Hsieh, Ho-Chang Kuo

**Affiliations:** ^1^ Kawasaki Disease Center, Kaohsiung Chang Gung Memorial Hospital, Kaohsiung, Taiwan; ^2^ Graduate Institute of Clinical Medical Sciences, College of Medicine, Chang Gung University, Kaohsiung, Taiwan; ^3^ Department of Pediatrics, Kaohsiung Chang Gung Memorial Hospital and Chang Gung University College of Medicine, Kaohsiung, Taiwan; ^4^ Genomics and Proteomics Core Laboratory, Department of Medical Research, Kaohsiung Chang Gung Memorial Hospital and Chang Gung University College of Medicine, Kaohsiung, Taiwan; ^5^ Department of Otolaryngology, Kaohsiung Chang Gung Memorial Hospital and Chang Gung University College of Medicine, Kaohsiung, Taiwan

**Keywords:** coronary artery lesions, FcγRIIA, FcγRIIB, intravenous immunoglobulin, Kawasaki disease, Immunology and Microbiology Section, Immune response, Immunity

## Abstract

Previous research has found patients with the *FcγRIIIB NA1* variant having increased risk of intravenous immunoglobulin (IVIG) resistance in Kawasaki disease (KD). Our previous studies revealed that elevated *FcγRIIA* expression correlated with the susceptibility of KD patients. We conducted this research to determine whether and how Fcγ receptors affect the susceptibility, IVIG treatment response, and coronary artery lesions (CAL) of KD patients. The activating *FcγRIIA* and inhibitory *FcγRIIB* methylation levels of seven patients with KD and four control subjects were examined using HumanMethylation27 BeadChip. We enrolled a total of 44 KD patients and 10 control subjects with fevers. We performed real-time RT-PCR to determine the *FcγRIIA* and *FcγRIIB* expression levels, as well as a luciferase assay of *FcγRIIA*. We found a considerable increase in methylation of both *FcγRIIA* and *FcγRIIB* in KD patients undergoing IVIG treatment. Promoter methylation of *FcγRIIA* inhibited reporter activity in K562 cells using luciferase assay. The *FcγRIIB* mRNA expression levels were not found to increase susceptibility, CAL formation, or IVIG resistance. *FcγRIIA* mRNA expression levels were significantly higher in IVIG-resistant patients than in those that responded to IVIG during the pre-treatment period. Furthermore, the *FcγRIIA/IIB* mRNA expression ratio was considerably higher in KD patients with CAL than in those without CAL. *FcγRIIA* and *FcγRIIB* both demonstrated increased methylation levels in KD patients that underwent IVIG treatment. *FcγRIIA* expression influenced the IVIG treatment response of KD patients. The *FcγRIIA/IIB* mRNA expression ratio was greater in KD patients with CAL formation.

## INTRODUCTION

A type of vasculitis, Kawasaki disease (KD) occurs most commonly in children, surpassing acute rheumatic fever as their leading cause of acquired heart disease [1]. KD is an acute multi-system vasculitis syndrome that consists of a prolonged fever and at least four of the following five diagnostic criteria: polymorphous rash, non-exudative conjunctivitis, oral-mucosal involvement, extremity desquamation, and cervical lymphadenopathy. The immunopathogenesis of KD is complex and not yet completely understood. Immune complexes are occasionally found in the peripheral blood of KD patients [2]. Genetic mapping and candidate gene analyses have indicated that KD is correlated with a variety of chromosomal regions, as well as several candidate genes that control susceptibility of KD, coronary aneurysm formation, and intravenous immunoglobulin (IVIG) treatment responsiveness [3]. A diagnosis of Kawasaki disease must be considered for any child with a prolonged fever of unknown origin. For KD treatment, a single high dose of IVIG is highly effective for lowering both the fever and the incidence of coronary aneurysms. However, IVIG's pharmacological mechanisms are also not fully understood. Treatments have been proposed based on the immune suppressive action of IVIG, such as stimulating the upregulation of inhibitory Fcγ receptors on macrophages and competing with immunocomplexes to activate Fcγ receptors [4]. In a study of mice with inflammatory arthritis, IVIG with glycans terminating in α2,6 sialic acids attached to the lectin dendritic-cell-specific ICAM-3 grabbing non-integrin (DC-SIGN) and promoted the creation of IL-33, which then expanded the IL-4+ basophils. These Th2 cytokines can inhibit autoantibody-mediated inflammation by modulating *FcγRIIB* expression on effector cells [5]. A number of Fcγ functions are maintained among various species. In humans, IVIG treatment inhibits dendritic cell function through the Th2 cytokine-mediated (IL-4 and IL-13) downregulation of *FcγRIIA* and *IFN-γR2*, not through the upregulation of *FcγRIIB* [6]. Th2 cells are critical for host protection against multicellular parasites, such as intestinal helminthes; when such cells are deregulated, they can contribute to such atopic diseases as asthma and enhance forms of vasculitis like eosinophilic granulomatosis with polyangiitis and inflammatory arthritis [5, 7]. Previous research has indicated that Th2 cells are crucial in KD's pathogenesis. Elevated eosinophil levels after IVIG treatment may be correlated with IVIG responsiveness [8]. We have demonstrated that Th2 immune-related responses (eosinophils, IL-4, IL-5, eotaxin, and eosinophil cationic protein) correlated with the susceptibility to KD and disease outcomes, as well as that interleukin-31, which is known to be related to Th2 cytokines, correlated with coronary artery lesions (CAL) when compared to the febrile control subjects [9, 10]. Genome-wide association studies and linkage analyses of KD have found that genes that contributed to eosinophil degranulation (a functional polymorphism in the IgG receptor gene *FcγRIIA*) correlated with KD [11]. Furthermore, an increased risk for atopic dermatitis was found in preschool children with KD in Taiwan [12]. We also found a positive correlation between KD and the risk of allergic diseases, which agrees with the findings of previous research [13].

Specific *FcγR* polymorphisms have been determined to be associated with the induction or severity of KD and patient responsiveness to IVIG [14]. However, each of these genetic associations is limited by the unequal polymorphic variations among the various ethnic groups studied. The FcγRIIA-H131 variant correlates with KD, while KD patients’ responsiveness to IVIG therapy is strongly associated with the *FcγRIIIB* genotype; the NA1 variant significantly reduces the probability of a proper clinical outcome [15]. Likewise, the lower copy number of*FcγRIIC* has been correlated with KD susceptibility [16].

These results indicate that F*cγ* receptors may be related to KD's clinical and pathological features; however, not much is known about their functional relationship. To better understand this phenomenon, we measured Fcγ receptors’ (activating *FcγRIIA* and inhibitory *FcγRIIB*) mRNA levels in KD patients and investigated the clinical importance of Fcγ receptors. Previous studies have found that epigenetic regulatory mechanisms correlate with a predisposition to KD and IVIG resistance [17]. The current study researched the DNA methylation of *FcγRIIA* and *FcγRIIB* using HumanMethylation27 BeadChip (Illumina, San Diego, CA, USA) in KD patients. A pyrosequencing assay was used to carry out confirmation with another cohort of DNA methylation array. We further evaluated the functional properties of the *FcγRIIA* promoter CpG methylation using a luciferase assay.

## RESULTS

### Demographic data

We recruited a total of 44 patients with Kawasaki disease (1.52 ± 0.17 years old, 21 male) for this case-control study (Table 1). Another 10 patients with an acute febrile infectious disease (1.95 ± 0.84 years old, 6 male) were selected as control subjects. The acute infections among the control group were upper or lower respiratory tract infections or gastroenteritis. No significant difference in age or gender was found between the KD patients and the control group. Of all the participants, seven patients (15.9%) had CAL formation, and four patients (9%) were IVIG resistant.

**Table 1:** Demographic data of Kawasaki disease patients and control subjects

	Controls	Kawasaki disease	*p*-value
mRNA study	(*N* = 10)	(*N* = 44)	
Age (year)	1.95 ± 0.84	1.52 ± 0.17	0.268
Male	6 (60%)	21 (47.8%)	0.484
Clinical data	Upper respiratory tract infection (*N* = 2) Lower respiratory tract infection (*N* = 6) Gastroenteritis (*N* = 2)	IVIG resistance: 4 patients (9 %) CAL: 7 patients (15.9 %)	
Pyrosequencing	(*N* = 54)	(*N* = 42)	
Age <5 years	100 %	100 %	
Male	31 (57.4%)	27 (64.3%)	0.534

a) Data are shown as mean ± standard error.

b) Abbreviations: CAL, coronary artery lesions; IVIG, intravenous immunoglobulin

### *FcγRIIA* and *FcγRIIB* expression and CAL formation

**Figure 1:**
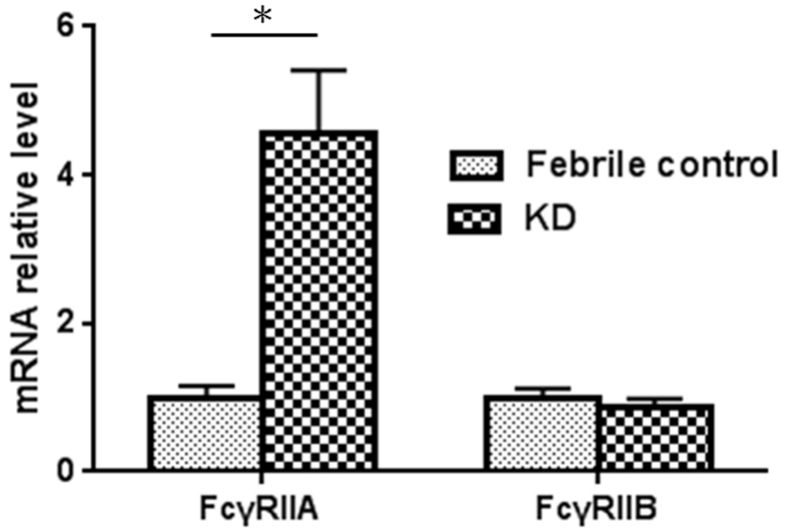
*FcγRIIA* and *FcγRIIB* mRNA expression in KD patients and control subjects (KD patients, *N* = 44; control, *N* = 10) *A *p*-value <0.05 between the two groups.

**Figure 2:**
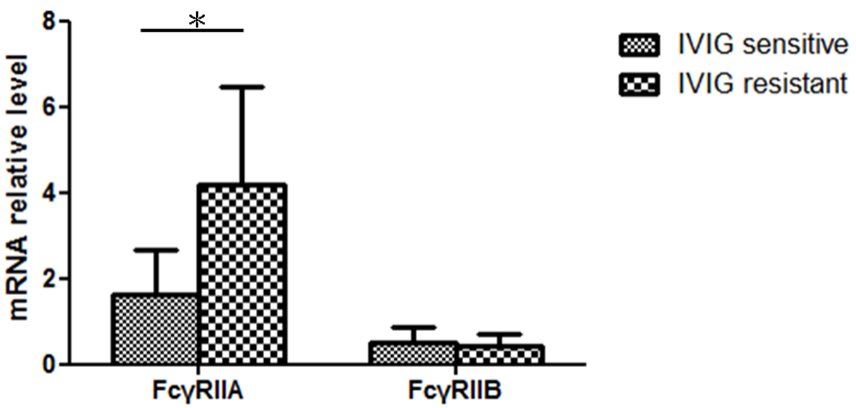
Considerably higher *FcγRIIA* mRNA levels in IVIG-resistant KD patients (IVIG resistant *N* = 4; IVIG sensitive, *N* = 40)

In the expression analysis, our data is presented as normalized to the mRNA levels of the control group patients. In Figure 1, the *FcγRIIA* and *FcγRIIB* values are shown normalized to the febrile controls. In our previous research, an increase in *FcγRIIA* mRNA levels was discovered in KD patients [17], but we found no significant differences between the KD patients and the febrile controls with regard to the mRNA levels of *FcγRIIB in this study.* In a previous report, we observed a considerable decrease in *FcγRIIA* mRNA expression after KD patients received IVIG treatment [17]. During the study period, *FcγRIIB* expression gradually reduced after IVIG treatment, although it was not statistically significant. No significant differences were found with regard to *FcγRIIA* and *FcγRIIB* mRNA levels between KD patients with or without CAL before and after IVIG therapy. The *FcγRIIA* expression varied considerably between the KD patients that responded to IVIG treatment and those that were IVIG resistant (*p* = 0.025) (Figure 2). On the other hand, we observed no significant difference between the mRNA levels of *FcγRIIB* the IVIG-resistant patients and the IVIG–responsive patients.

**Figure 3:**
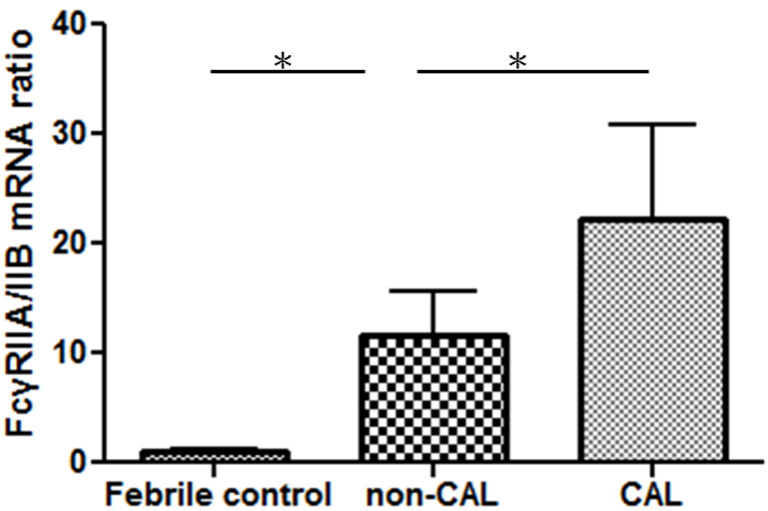
The *FcγRIIA* and *FcγRIIB* mRNA expression ratio of KD patients with (*N* = 7) and without CAL (*N* = 37) and the control subjects (*N* = 10) was determined using quantitative PCR *p-value < 0.05.

Compared to the febrile controls, KD patients demonstrated a considerably increased *FcγRIIA/IIB* mRNA ratio (13.29 ± 3.72 vs. 1.01 ± 0.14; *P* < 0.001). The *FcγRIIA/IIB* mRNA expression ratio was also significantly higher in patients with CAL when compared to those without CAL (22.22 ± 8.56 vs. 11.60 ± 4.10; *p* = 0.007) (Figure 3). These findings indicate a shifting balance of the two opposing *FcγR* isoforms toward an activating phenotype in KD patients and patients with CAL.

### Methylation levels of *FcγRIIA* and *FcγRIIB*

Previous evidence has indicated that epigenetic changes may offer an alternate explanation of KD's pathophysiology. We performed additional analysis of methylation with the Illumina HumanMethylation27 BeadChip array in order to explore the methylation profiles of *FcγRIIA* and *FcγRIIB* that could account for the disease's clinical pathophysiology. We recruited seven children with KD, as well as four febrile children as controls, for this study. We carried out microarray analysis with the HumanMethylation27 BeadChip on genomic DNA from the entire blood, finding significant hypomethylation of the *FcγRIIA* (cg24422489) promoters, which are functionally associated with genes discovered by prior studies to be differentially methylated in KD patients [17]. In this study, we found that *FcγRIIA* (cg27470554) and *FcγRIIB* (cg22436411) methylation levels were not lower in KD patients than in the febrile controls (*p* = 0.387 and *p* = 0.957, respectively) (Table 2). HumanMethylation27 *FcγRIIA* (cg27470554 and cg24422489, *p* = 0.002 and 0.002 respectively) and *FcγRIIB* (cg22436411, *p* = 0.013) demonstrates noticeably higher methylation levels after patients underwent IVIG treatment (Table 2). To verify the methylation array results, we utilized target-specific sequencing at cg22436411 in the *FcγRIIB* promoter region through pyrosequencing, which resulted in only one methylation site. With an additional 42 KD patients and 54 controls, the methylation corroboration showed that this *FcγRIIB* hypomethylation difference did not achieve statistical significance.

**Table 2:** Significant increase in methylation of *FcγRIIA* and *FcγRIIB* after intravenous immunoglobulin treatment

Target ID	Symbol	FC *N* = 4	KD1 *N* = 7	KD-3 *N* = 7	*p*-value (KD1*vs*. FC)	*p*-value (KD1*vs*. KD3)
cg22436411	FcγRIIB	0.070 ± 0.043	0.062 ± 0.041	0.132 ± 0.063	0.957	0.013*
cg27470554	FcγRIIA	0.052 ± 0.013	0.046 ± 0.015	0.072 ± 0.011	0.387	0.002**
cg24422489	FcγRIIA	0.620 ± 0.127	0.465 ± 0.167	0.727 ± 0.129	0.007*	0.002**

a) Data are shown as mean ± standard deviation.

b) We tested p-values using the independent sample *t*-test between FC and KD1; we tested p-values using the paired *t*-test between KD1 and KD3.

* c) indicating p-value < 0.05. ;

** indicating *p*-value < 0.005.

d) Abbreviations: FC, febrile control; KD1, Kawasaki disease before intravenous immunoglobulin; KD3, at least three weeks after intravenous immunoglobulin.

### Functional analysis of the *FcγRIIA* promoter

Using both the HumanMethylation27 BeadChip array and pyrosequencing methods, we observed *FcγRIIA* promoter hypomethylation in KD patients. Due to the luciferase assay, we decided to research whether DNA methylation could regulate *FcγRIIA* expression instead of using CpG sites mutation [17]. To establish a direct link between the *FcγRIIA* expression levels and their methylation state, we performed reporter gene analysis that included cloning the *FcγRIIA* promoter region.

**Figure 4:**
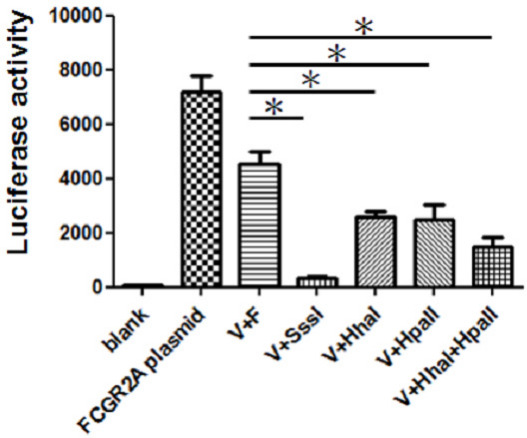
Comparative analysis of differential promoter methylation *In vitro* methylation inhibits *FcγRIIA* promoter reporter activity in K562 cells. (*p < 0.05). All data are representative of at least six experiments. The transfection of K562 cells was performed with LightSwitch vector and *FcγRIIA* promoter plasmid (V+F) or vector (V) treated with methyltransferase SssI, Hha I, or Hpa II methylases.

The CpG methylase Sss I-treated and untreated plasmids were transfected into K562 cells. As shown in Figure 4, the results from the luciferase experiments, which were repeated at least six times, demonstrate that promoter activity was distinctly influenced by CpG methylation. These findings indicate that both of the CpG-containing reporter vectors were successfully inhibited through DNA methylation by CpG methylase Sss I. To determine whether partial methylation may also influence reporter activity, the plasmids were either methylated using site-specific methylases Hha I, and/or Hpa II or were left unmethylated. Luciferase activity was assayed in K562 cells once transient transfection was completed. Figure 4 shows that the reporter activity of the differentially methylated plasmids significantly decreased.

## DISCUSSION

The current study has found that treating KD patients with IVIG can considerably reduce the elevated mRNA levels of *FcγRIIA*. Our previous research validated *FcγRIIA* methylation [17]. The present results which find *FcγRIIA* expression to be significantly higher in IVIG-resistant patients support our previous study that shows that KD with IVIG resistance demonstrated hypomethylation [17]. Furthermore, we recently discovered a significant correlation among the promoter methylation of *FcγRIIA*, susceptibility of Kawasaki disease, and therapeutic outcomes of IVIG treatment [18]. Through functional testing, we discovered that *in vitro* methylation of the *FcγRIIA* promoter decreased luciferase activity. Elevated F*cγRIIA/FcγRIIB* expression has also been seen in patients with CAL; therefore, the ratio is altered to favor cell activation.

Human Fc gamma receptors (FcγRs), which are the receptors for the Fc region of immunoglobulinG (IgG) antibodies, are vital in this process as they control the production of tissue- and pathogen-specific cytokines [19]. FcγRs are responsible for such processes as uptake, antigen presentation, and antibody dependent cellular cytotoxicity [19]. Inhibitory FcγRIIB receptors are primarily expressed by B cells, monocytes, and macrophages and are not probable factors to explain IVIG's immunomodulatory capacity [20]. *FcγRIIB* influences humans’ susceptibility to several autoimmune diseases, including systemic lupus erythematosus, rheumatoid arthritis, anti-GBM disease, and idiopathic thrombocytopenic purpura [21]. Genes have functional polymorphisms that can manage these receptors to alter the balance between activation and inhibition and ultimately their interaction. Elevated *FcγRIIA/IIB* mRNA expression levels in untreated KD patients reflect the potential role of a disrupted FcγR balance in the pathogenesis of CAL formation. Such expression variations may, at least in part, occur secondary to inflammation. IVIG responses in KD patients depend on *FcγRIIB* polymorphisms in a racially dependent manner [22]. The Asian individuals studied lacked these SNPs, which agrees with our reports that found that the *FcγRIIB* mRNA expression levels did not appear to affect KD susceptibility, CAL formation, or IVIG resistance. We performed independent validation of methylation profiles by analyzing *FcγRIIB* data from clinical samples of patients’ blood. Pyrosequencing is superior to methylation-specific PCR due to such advantages as requiring only a small amount of DNA, flexibility, accuracy, and quantification [23].

Various studies have confirmed a significant relationship between *FcγRIIA* and KD. Elevated *FcγRIIA* expression has been observed in KD patients [17], and the epigenetic dysregulation of *FcγRIIA* is vital for facilitating the disease's clinical outcome [18]. Furthermore, IVIG treatment can potentially alter DNA methylation/demethylation dynamics. This data shows that IVIG treatment can increase the DNA methylation of *FcγRIIA* and *FcγRIIB*. As for the functional significance of *FcγRIIA* methylation, which we determined by testing the influence of promoter methylation *in vitro*, our experimental data clearly shows that methylation of the *FcγRIIA* promoter can control luciferase activity in K562 cells.

As described in our previous study of *FcγRIIA* mRNA and KD, functional studies have been performed using CpG sites’ point mutation with pGL3-Basic vector in cultured Chang liver cells [17]. Unlike the present study, that study focused specifically on the regional CpG site of cg24422489 instead of the whole promoter. In this study, luciferase assay with reporter vector (LightSwitch) in K562 cells also revealed that methylation of the *FcγRIIA* promoter using methyltransferase significantly decreased promoter activity. However, methylation cannot be precisely targeted to individual single CpGs as site-specific intervention using this method. The findings of both of the functional luciferase studies strongly suggest that methylation of the *FcγRIIA* promoter could significantly reduce gene expression.

This study has some limitations. First, we did not research the genetic variants in *FcγRIIA* that may also alter the variability of the *FcγRIIA* expression. We also did not assess other expression levels of FcγR or the expression levels of different blood cells.

The present study's results confirm that *FcγRIIA* mRNA was upregulated in KD patients with IVIG resistance. We also determined that DNA methylation in the *FcγRIIA* promoter region was the fundamental mechanism for the expression differences of *FcγRIIA* in KD, which was verified through *in vitro* cell culture experiments. Furthermore, our data indicates that *FcγRIIA/FcγRIIB* expression is elevated in patients with CAL. Therefore, *FcγRIIA* can be considered a valuable marker for predicting KD's treatment outcome. Our discovery that *FcγRIIA* methylation influences epigenetic regulators reinforces the importance of such changes in KD's pathophysiology and emphasizes the potential of epigenetic modulators for successfully treating KD.

## MATERIALS AND METHODS

### Subjects

This study features 44 patients diagnosed with KD, as well as 10 febrile, age- and gender-matched control subjects. We took peripheral blood samples at three points during the study: before IVIG treatment (KD1), shortly after IVIG treatment (within three days, KD2), and at least three weeks after IVIG treatment (KD3), and then analyzed *FcγRIIA* and *FcγRIIB* mRNA levels using a quantitative real-time polymerase chain reaction. Subjects were diagnosed with KD by a clinician using the recommended universal KD criteria proposed by the American Heart Association (AHA) [24]. KD was diagnosed in any child with a fever that lasts longer than five days and manifested four of the following five criteria: diffuse mucosal inflammation with strawberry tongue and fissure lips, bilateral nonpurulent conjunctivitis, indurative angioedema of the hands and feet, dysmorphic skin rashes, and unilateral cervical lymphadenopathy [25]. Pursuant to the guidelines of the American Academy of Pediatrics and the American Heart Association, KD patients were treated with high doses of IVIG (2 g/kg) within 12 h of diagnosis [24]. IVIG responsiveness (IVIG sensitivity) is defined as fever reduction within 48 hours after initial IVIG treatment and no reoccurrence of fever (>38°C) for seven or more days. We used two-dimensional echocardiography to identify the presence of CAL, which are defined as coronary arteries with a diameter larger than 3 mm (or 4 mm in patients older than five years). Among the participants, seven developed CAL. This study was approved by Chang Gung Memorial Hospital's Internal Review Board, and we obtained written informed consent from the parents or guardians of all the participating children.

### Confirming DNA methylation array using pyrosequencing assay

In our previous studies, we carried out DNA methylation array using the HumanMethylation27 BeadChip and then pyrosequencing assay to confirm the DNA methylation array. A total of 500 ng genomic DNA was treated with bisulfite using an EZ DNA methylation kit (ZYMO Research) in accordance with the manufacturer's instructions. It was then washed in 20 μl of Tris-Buffer (10 mM). All primers were specifically designed for bisulfite-converted DNA, which was amplified by PCR using unbiased nested primers. PCR was performed in a 25-μl reaction mixture of 25 ng of bisulfite-converted DNA, 1X Pyromark PCR Master Mix (Qiagen, Valencia, CA, USA), and 0.2 μM *FcγRIIB* biotinylated forward primer 5’-ATGTTGAGGGTGAATAAATGG-3’ and reverse primer 5’-biotin-ATATTCCCCAAAAAATAAATTACCCCTAAC-3’ with the following PCR program: 95°C for 5 min, then 45 cycles of 95°C for 30 s, followed by 55°C for 30 s, and 72°C for 30 s, with a final extension of 72°C for 5 min. After amplification, the biotinylated PCR products were purified and incubated with the sequencing primer 5’-GGTAATGAGGATGATGATA-3’, which was created** to be attached to the CpG sites of interest. We evaluated DNA methylation with quantitative pyrosequencing using a PyroMark Q24 instrument (Qiagen, Valencia, CA, USA) and** established the DNA methylation percentage at each CpG site using PyroMark Q24 1.010 software.

### Real-time RT-PCR of *FcγRIIA* and *FcγRIIB*

All RNA was isolated from white blood cells using the FavorPrep Blood/Cultured Cell Total RNA Purification Kit (Favorgen, cat. No. FABRK001-1). We utilized the high-performance reverse-transcriptase system (EPICENTRE) to perform reverse transcription of the RNA samples. Both *FcγRIIA* and *FcγRIIB* expression levels were detected with real-time RT-PCR using the SYBR Green PCR Master Mix and ABI Prism 7500 Sequence Detection System (Applied Biosystems). The primers used to amplify *FcγRIIA* mRNA were 5’-TCA TTG TGG CTG TGG TCA TT G-3’(forward) and 5’-CCT GGG GTT CAG AGT CAT GT-3’(reverse); those for amplifying *FcγRIIB* mRNA were 5’GTT GGG GCT GAG AAC ACA AT 3’ (forward) and 5’ACA GGG AGC TTC AGG ACT CA 3’ (reverse).

### Luciferase assay to functionally analyze FcγRIIA promoter CpG methylation

To investigate *FcγRIIA* promoter activity, the human *FcγRIIA* promoter-reporter luciferase construct commercially synthesized by LightSwitch was processed using the restriction enzymes Mlu I and Bgl II; said digestion process was then confirmed with agarose gel ectrophoresis. We treated 5 ug plasmids with non-specific CpG methyltransferase SssI (New England BioLabs), Hha I, Hpa II methylases, and Hha I + Hpa II for 4 hours in accordance with the manufacturer's instructions, and the change of CpG was analyzed with plasmid DNA bisulfite modification and pyrosequencing using specific primers (Pyromark ID96 system, Biotage). The methylated plasmids were ligated into the luciferase vector. The aforementioned inserted fragments and genes were confirmed using DNA sequencing. The human immortalized myelogenous leukemia cell line K562 *FcγRIIA*in accordance with the manufacturer's instructionstransfection of the plasmids in K562 cells was performed with Lipofectamine 2000 (Invitrogen).Once transfected for 24 hours, using a Dual-Luciferase Reporter Assay System (Promega). We used the un*FcγRIIA* as the control.

### Statistics

The data in this study are expressed as mean ± standard error. We used student's t-test to compare demographic data and the Mann–Whitney U-test to compare mRNA expression levels. Any differences in the blood's measured variables before and after IVIG treatment were pairwise analyzed using the Friedman test. A p-value less than 0.05 was considered statistically significant. All statistical tests were carried out with SPSS 14.0 for Windows XP (SPSS, Inc., Chicago, IL, USA).

## References

[1] Kuo HC, Yang KD, Chang WC, Ger LP, Hsieh KS (2012). Kawasaki disease: an update on diagnosis and treatment. Pediatr Neonatol.

[2] Mason WH, Jordan SC, Sakai R, Takahashi M, Bernstein B (1985). Circulating immune complexes in Kawasaki syndrome. Pediatr Infect Dis.

[3] AH R (2011). Kawasaki disease: novel insights into etiology and genetic susceptibility. Annu Rev Med.

[4] Chong BH, C J (2010). IVIg immune inhibitory activity: APC is key. Blood.

[5] Anthony RM, K T, Wermeling F, Ravetch JV (2011). Intravenous gammaglobulin suppresses inflammation through a novel T(H)2 pathway. Nature.

[6] Tjon AS, van Gent R, Jaadar H, Martin van Hagen P, Mancham S, van der Laan LJ, te Boekhorst PA, Metselaar HJ, Kwekkeboom J (2014). Intravenous immunoglobulin treatment in humans suppresses dendritic cell function via stimulation of IL-4 and IL-13 production. J Immunol.

[7] Vaglio A, Moosig F, Zwerina J (2012). Churg-Strauss syndrome: update on pathophysiology and treatment. Curr Opin Rheumatol.

[8] Kuo HC, Yang KD, Liang CD, Bong CN, Yu HR, Wang L, Wang CL (2007). The relationship of eosinophilia to intravenous immunoglobulin treatment failure in Kawasaki disease. Pediatr Allergy Immunol.

[9] Tseng WN, Lo MH, Guo MM, Hsieh KS, Chang WC, Kuo HC (2014). IL-31 associated with coronary artery lesion formation in Kawasaki disease. PLoS One.

[10] Kuo HC, Wang CL, Liang CD, Yu HR, Huang CF, Wang L, Hwang KP, Yang KD (2009). Association of lower eosinophil-related T helper 2 (Th2) cytokines with coronary artery lesions in Kawasaki disease. Pediatr Allergy Immunol.

[11] Khor CC, Davila S, Breunis WB, Lee YC, Shimizu C, Wright VJ, Yeung RS, Tan DE, Sim KS, Wang JJ, Wong TY, Pang J, Mitchell P (2011). Genome-wide association study identifies FCGR2A as a susceptibility locus for Kawasaki disease. Nat Genet.

[12] Woon PY, Chang WC, Liang CC, Hsu CH, Klahan S, Huang YH, Chang WP, Kuo HC (2013). Increased risk of atopic dermatitis in preschool children with kawasaki disease: a population-based study in taiwan. Evid Based Complement Alternat Med.

[13] Kuo HC, Chang WC, Yang KD, Yu HR, Wang CL, Ho SC, Yang CY (2013). Kawasaki disease and subsequent risk of allergic diseases: a population-based matched cohort study. BMC Pediatr.

[14] Gillis C, Gouel-Cheron A, Jonsson F, Bruhns P (2014). Contribution of Human FcgammaRs to Disease with Evidence from Human Polymorphisms and Transgenic Animal Studies. Front Immunol.

[15] Shrestha S, Wiener H, Shendre A, Kaslow RA, Wu J, Olson A, Bowles NE, Patel H, Edberg JC, Portman MA (2012). Role of activating FcgammaR gene polymorphisms in Kawasaki disease susceptibility and intravenous immunoglobulin response. Circ Cardiovasc Genet.

[16] Makowsky R, Wiener HW, Ptacek TS, Silva M, Shendre A, Edberg JC, Portman MA, Shrestha S (2013). FcgammaR gene copy number in Kawasaki disease and intravenous immunoglobulin treatment response. Pharmacogenet Genomics.

[17] Kuo HC, Chang JC, Yu HR, Wang CL, Lee CP, Huang LT, Yang KD (2015). Identification of an association between genomic hypomethylation of FCGR2A and susceptibility to Kawasaki disease and intravenous immunoglobulin resistance by DNA methylation array. Arthritis Rheumatol.

[18] Kuo HC, Hsu YW, Wu MS, Woon PY, Wong HS, Tsai LJ, Lin RK, Klahan S, Hsieh KS, Chang WC (2015). FCGR2A Promoter Methylation and Risks for Intravenous Immunoglobulin Treatment Responses in Kawasaki Disease. Mediators Inflamm.

[19] Vogelpoel LT, Baeten DL, de Jong EC, den Dunnen J (2015). Control of cytokine production by human fc gamma receptors: implications for pathogen defense and autoimmunity. Front Immunol.

[20] Nagelkerke SQ, Kuijpers TW (2014). Immunomodulation by IVIg and the Role of Fc-Gamma Receptors: Classic Mechanisms of Action after all?. Front Immunol.

[21] Smith KG, Clatworthy MR (2010). FcgammaRIIB in autoimmunity and infection: evolutionary and therapeutic implications. Nat Rev Immunol.

[22] Shrestha S, Wiener HW, Olson AK, Edberg JC, Bowles NE, Patel H, Portman MA (2011). Functional FCGR2B gene variants influence intravenous immunoglobulin response in patients with Kawasaki disease. J Allergy Clin Immunol.

[23] Quillien V, Lavenu A, Ducray F, Joly MO, Chinot O, Fina F, Sanson M, Carpentier C, Karayan-Tapon L, Rivet P, Entz-Werle N, Legrain M, Zalcman EL (2016). Validation of the high-performance of pyrosequencing for clinical MGMT testing on a cohort of glioblastoma patients from a prospective dedicated multicentric trial. Oncotarget.

[24] Newburger JW, Takahashi M, Gerber MA, Gewitz MH, Tani LY, Burns JC, Shulman ST, Bolger AF, Ferrieri P, Baltimore RS, Wilson WR, Baddour LM, Levison ME (2004). Diagnosis, treatment, and long-term management of Kawasaki disease: a statement for health professionals from the Committee on Rheumatic Fever, Endocarditis and Kawasaki Disease, Council on Cardiovascular Disease in the Young, American Heart Association. Circulation.

[25] Kuo HC, Hsu YW, Wu MS, Chien SC, Liu SF, Chang WC (2016). Intravenous immunoglobulin, pharmacogenomics, and Kawasaki disease. Journal of microbiology, immunology, and infection.

